# Test-Retest Variability of Relative Tracer Delivery Rate as Measured by [^11^C]PiB

**DOI:** 10.1007/s11307-021-01606-z

**Published:** 2021-04-21

**Authors:** Fiona Heeman, Janine Hendriks, Isadora Lopes Alves, Nelleke Tolboom, Bart N. M. van Berckel, Maqsood Yaqub, Adriaan A. Lammertsma

**Affiliations:** 1 Amsterdam UMC, Vrije Universiteit Amsterdam, Radiology and Nuclear Medicine, Amsterdam Neuroscience, De Boelelaan 1117, Amsterdam, The Netherlands; 2 Imaging Division, Department of Radiology, University Medical Center Utrecht, Utrecht, Netherlands

**Keywords:** [^11^C]PiB, Alzheimer’s disease, Cerebral blood flow, Relative tracer delivery, Test-retest variability

## Abstract

**Purpose:**

Moderate-to-high correlations have been reported between the [^11^C]PiB PET-derived relative tracer delivery rate *R*_1_ and relative CBF as measured using [^15^O]H_2_O PET, supporting its use as a proxy of relative CBF. As longitudinal PET studies become more common for measuring treatment efficacy or disease progression, it is important to know the intrinsic variability of *R*_1_. The purpose of the present study was to determine this through a retrospective data analysis.

**Procedures:**

Test-retest data belonging to twelve participants, who underwent two 90 min [^11^C]PiB PET scans, were retrospectively included. The voxel-based implementation of the two-step simplified reference tissue model with cerebellar grey matter as reference tissue was used to compute *R*_1_ images. Next, test-retest variability was calculated, and test and retest *R*_1_ measures were compared using linear mixed effect models and a Bland-Altman analysis.

**Results:**

Test-retest variability was low across regions (max. 5.8 %), and test and retest measures showed high, significant correlations (*R*^2^=0.92, slope=0.98) and a negligible bias (0.69±3.07 %).

**Conclusions:**

In conclusion, the high precision of [^11^C]PiB *R*_1_ suggests suitable applicability for cross-sectional and longitudinal studies.

## Background

Cerebral blood flow (CBF) is known to decline with age, and elderly individuals (75–80 years) may present with reductions in CBF of up to 25 % compared with young adults (±25 years) [1, 2]. In the context of Alzheimer’s disease (AD), additional reductions in CBF have been reported in several cortical brain regions such as the frontal, parietal and temporal cortices with both absolute reductions as well as relative to cerebellar grey matter reference tissue [3, 4]. This pattern of CBF reductions is considered characteristic of AD pathology and may therefore be used as proxy for measuring disease severity or progression [5, 6]. The gold standard technique for measuring CBF is [^15^O]H_2_O positron emission tomography (PET) [7], but MR-based techniques such as arterial spin labelling (ASL) have also been introduced [5]. More recently, several studies have evaluated whether a valid proxy of cerebral perfusion can be obtained from the early frames of dynamic scans using currently available PET tracers (e.g. for measuring amyloid-β or tau burden) [3, 8]. The relative influx rate (*R*_1_=*K*_1_/*K*_1_’), which can be calculated from these early frames, is an indirect measure of relative CBF as it is also affected by the extraction fraction (*K*_1_=*E*·*CBF*). In particular, for the amyloid tracer [^11^C]PiB, Chen and colleagues have reported that in cortical regions, relative tracer delivery *R*_1_ showed moderate-to-high correlations with relative CBF measured using dynamic [^15^O]H_2_O PET (with a range of *ρ*=0.68–0.84, *p*<0.001, across cortical regions and a value of *ρ*=0.82, *p*<0.001, for the global cortical region), thereby indicating that [^11^C]PiB could be used for dual-biomarker imaging [9]. Furthermore, Bilgel and colleagues demonstrated that in a longitudinal setting with an average follow-up duration of 2.5 years, rates of change as measured with *R*_1_ showed low-to-moderate correlations with changes in [^15^O]H_2_O PET CBF (median *r* =0.42 across cortical regions and a value of *r* =0.65 for the global cortical region). In addition, rates of change as measured with *R*_1_ required the smallest sample-size for detecting group-wise differences (27 % reduction compared with [^15^O]H_2_O PET), suggesting this proxy could be suitable for tracking long-term longitudinal changes in CBF [8]. As longitudinal PET studies in AD become more common for measuring treatment efficacy or disease progression, it is important to know the intrinsic variability of *R*_1_ in order to determine what magnitude of change in *R*_1_ signifies an actual change. Therefore, the purpose of the present retrospective analysis of a previously reported test-retest (TRT) [^11^C]PiB study was to assess the precision of [^11^C]PiB *R*_1._

## Materials and Methods

### Subjects

Data from twelve participants belonging to a TRT study conducted within the Amsterdam UMC, location VUmc, were reanalysed as the original study only reported TRT variability for the non-displaceable binding potential [10]. This dataset consisted of five cognitively unimpaired (CU) subjects, one patient with mild cognitive impairment (MCI) and six with AD dementia, which in the present study was used to examine TRT variability for *R*_1_ [10]. Before enrolment, written informed consent was obtained from all individual participants included in the study, and the Medical Ethics Review Committee of the Amsterdam UMC, location VUmc, had approved the study.

### Image Acquisition

All subjects underwent a structural T1-weighted MR scan on a 1.5T Siemens Sonata scanner and, within 1 week, two 90-min dynamic [^11^C]PiB PET scans (test and retest) on a Siemens ECAT EXACT HR+ scanner [10]. Each dynamic scan consisted of 23 consecutive time frames (1×15, 3×5, 3×10, 2×30, 3×60, 2×150, 2×300, 7×600 s). All participants received an intravenous injection of, on average 332±70 MBq for test (353±26 for CUs, 138 for the MCI patient, and 342±66 for the AD dementia patients)and 376±43 MBq (355±37 for CUs, 368 for the MCI patient, and 393±47 for the AD dementia patients) for retest scans.

### Image Processing

Structural T1-weighted MR images were co-registered to their corresponding PET image segmented into grey matter (GM), white matter (WM) and cerebrospinal fluid (CSF) using PVE-lab software [11]. Next, volumes of interest (VOIs) were delineated based on the Hammers atlas and a reference tissue time–activity curve (TAC) of the cerebellar grey matter was extracted [12, 13].

### Parametric Analysis

The PPET software tool [14] with the voxel-based implementation of the two-step simplified reference tissue model (SRTM2), as validated for [^11^C]PiB, and cerebellar grey matter as reference tissue were used to compute relative tracer delivery (*R*_1_) images [15–17]. For SRTM2, *k*_2_' was determined across all voxels with a *BP*_ND_ higher than 0.05 by taking the median *k*_2_' from a first run using receptor parametric mapping (RPM) [18]. Regional *R*_1_ values were obtained by superimposing the following grey matter VOIs on the parametric images: medial and lateral anterior temporal lobe, posterior temporal lobe, superior, middle and inferior temporal gyrus, fusiform gyrus, parahippocampal and ambient gyrus, anterior and posterior cingulate gyrus, middle and orbitofrontal gyrus, gyrus rectus, inferior and superior frontal gyrus, pre- and post-central gyrus, superior parietal gyrus and the (infero)lateral remainder of the parietal lobe and a global cortical composite region (i.e. volume-weighted average across all target regions).

### Statistical Analysis

Statistical analyses were performed in R (version 4.0.3; R Foundation for Statistical Computing, Vienna, Austria). First, global *R*_1_ values were compared between CU and AD dementia groups using a non-parametric Mann-Whitney *U* test, separately for test and retest scans. Next, TRT variability was calculated for regional and global cortical *R*_1_ values according to Eq. 1, where *T* represents the estimate of *R*_1_ measured during test, and *R* the one measured during retest.

1 $$ TrT\  variability\ \left(\%\right)=\frac{\mid T-R\mid }{0.5\bullet \mid T+R\mid}\bullet 100 $$

In addition, a correlation analysis was used to assess the relationship between TRT variability and regional volume. Furthermore, to assess the relationship between test and retest *R*_1_ measures, linear mixed effect models (LME) were fitted and correlation coefficients were calculated using the nlme and MuMIn packages, respectively [19, 20]. Visual read (amyloid-β positive or negative) was used as a covariate and the analysis accounted for the within-subject correlation between regions. Finally, a Bland-Altman analysis was used to assess potential bias between test and retest *R*_1_ using the blandr package [21, 22].

## Results

Participant characteristics are shown in Table 1. Relative tracer delivery measures (*R*_1_) are reported in Table 2, with a significantly lower global *R*_1_ in AD dementia patients compared with CU participants, for both test and retest scans (*p* < 0.01).

**Table 1:** Subject demographics

	CU (*N=* 5)	MCI (*N=*1)	AD dementia (*N=*6)
Age	64.6 ±6.4	71.0	61.0 ±3.0
Females	60 %	100 %	17 %
VR positive	20 %	0 %	100 %
MMSE	29.8 ±0.4	28.0	20.7 ±2.0

*VR* visual read, *MMSE* mini mental state examination

Values are depicted as mean±SD, unless indicated otherwise

**Table 2:** Relative tracer delivery values by diagnostic group

	SRTM2-derived *R*_1_		
Diagnostic groups	Test	Retest	
**CU** (*N*=5)	0.93 ± 0.04	0.91 ± 0.03	
MCI (*N*=1)	0.91	0.91	
AD dementia (*N*=6)	0.82 ± 0.04	0.82 ± 0.03	

Values are depicted as mean±SD

Regional and global cortical TRT variability values are presented in Table 3. TRT variability for the global cortical composite was low (1.70 %), while the range of regional TRT variability showed slightly higher values (range: 1.52–5.78 %). Furthermore, there was a trend effect towards smaller TRT variability for larger regions (*R*^2^=0.14, *p*=0.09).

**Table 3:** Regional test-retest variability (%) of *R*_1_

Region*	SRTM2-derived *R*_1_
Global Cortex	1.70
Anterior temporal lobe medial part	2.77
Anterior temporal lobe lateral part	2.48
Parahippocampal and ambient gyri	3.02
Superior temporal gyrus	2.68
Middle and inferior temporal gyri	2.87
Fusiform gyrus	2.26
Insula	2.06
Lateral remainder of occipital lobe	2.08
Gyrus cinguli anterior part	1.52
Gyrus cinguli posterior part	3.44
Middle frontal gyrus	2.13
Posterior temporal lobe	2.11
Inferolateral remainder of parietal lobe	2.31
Precentral gyrus	1.71
Gyrus rectus	5.78
Orbitofrontal gyri	3.06
Inferior frontal gyrus	2.00
Superior frontal gyrus	2.08
Postcentral gyrus	1.79
Superior parietal gyrus	1.59
Lingual gyrus	2.60
Cuneus	1.76

*All values are average % TRT variability across *N*=11 subjects

**Fig. 1.**
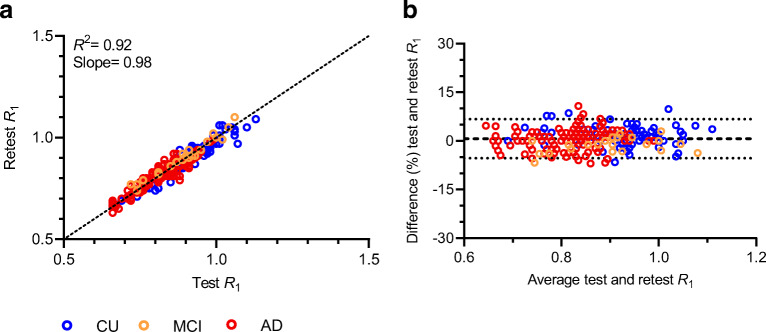
Relationship between SRTM2-derived test and retest *R*_1_. (**a**) The correlation between *R*_1_ test and retest measures, with *R*^2^ and slope parameters corresponding to the LME analysis and (**b**) a Bland-Altman plot, which indicates the bias between the two measures.

LME analyses showed that test and retest *R*_1_ values were strongly correlated and that the slope was not significantly different from 1 (*R*^2^=0.92, slope=0.98 C.I. [0.94–1.01], *p*<0.001). Furthermore, amyloid status as measured by visual read did not have a significant effect on this relationship. Finally, Bland-Altman analysis showed a negligible bias (0.69±3.07 %) between test and retest *R*_1_ (Fig. 1). All analyses were also carried out using RPM-derived *R*_1_ which resulted in essentially identical results (data not shown).

## Discussion

The present study assessed precision of the *R*_1_ parameter, a measure of relative tracer delivery, through a retrospective analysis of a previously reported [^11^C]PiB test-retest study [11]. Low test-retest variability was observed for SRTM2-derived *R*_1_, and this was true for regions of different sizes.

Differences in *R*_1_ between diagnostic groups were as expected, with lower average *R*_1_ values in AD dementia patients compared with CU participants. This finding is in agreement with existing literature where decreases in (relative) perfusion related to AD pathology have been reported for both *R*_1_ and [^15^O]H_2_O PET studies [3, 23, 24]. Furthermore, by incorporating these two groups, the present study covered the entire range of *R*_1_ values that would be expected in clinical studies across the AD spectrum.

Excellent TRT variability was observed for the global cortical composite (1.70 %) and only a slightly poorer TRT variability for some of the smaller regions (max 5.8 %). These findings were supported by the results of the LME analysis which showed a high correlation between test and retest *R*_1_ measures (*R*^2^=0.92) and a slope that was close to identity. As expected, the results indicate that smaller TRT variability was associated with larger regions. This finding suggests that studies should consider looking at relatively larger regions with PET when their aim is to detect small (<5 %) changes. Despite distinct kinetics, the present findings were also comparable with results from a [^18^F]florbetapir study that assessed TRT variability of SRTM-derived *R*_1_ in a very similar population in terms of age and diagnosis (max. TRT variability of 6 %) [25]. Comparing [^11^C]PiB *R*_1_ TRT variability with TRT variability of absolute perfusion as measured with the gold-standard, [^15^O]H_2_O PET [26], shows that the present results are slightly better, indicating that *R*_1_ is a more precise measure likely due to the fact that it is a relative measure. Furthermore, given that *R*_1_ is an indirect measure of perfusion, variation in the extraction fraction may compensate day-to-day fluctuations in flow to maintain constant delivery. On the other hand, this also means that alterations in extraction fraction may bias *R*_1_, as opposed to the gold standard [^15^O]H_2_O PET which provides a direct measurement of CBF. Unfortunately, to date, no studies have reported TRT variability of relative perfusion as measured with [^15^O]H_2_O PET with cerebellar grey matter reference tissue for a direct comparison. Nonetheless, a study by Bilgel and colleagues compared long-term variability of CBF proxies (i.e. [^11^C]PiB PET-derived *R*_1_ and early frame standardised uptake value ratios) to that of [^15^O]H_2_O PET and reported that, across these three measures, the highest longitudinal stability was obtained with *R*_1_. The present study demonstrates that *R*_1_ also has low short-term variability. Therefore, *R*_1_ could be considered a valid alternative to measuring longitudinal changes in CBF, thereby circumventing the need for a separate [^15^O]H_2_O PET scan. Unfortunately, it remains unclear whether changes across a lifetime are comparable between *R*_1_ and rCBF as measured by [^15^O]H_2_O PET. One study reports that, in an elderly population (77±8 years old), the yearly percentage change in *R*_1_ was lower (−0.28 %) than that in regional CBF (−0.41 %) as measured by [^15^O]H_2_O PET, although it was not reported whether this difference was significant [8]. Nevertheless, a smaller change in *R*_1_, especially in elderly subjects, may be related to an increased extraction fraction [27]. However, longitudinal studies of *R*_1_ in a younger population are needed to confirm whether the same difference is present earlier in life. Furthermore, using a relative parameter to measure CBF such as *R*_1_ essentially assumes that there are no CBF changes in the reference tissue (*R*_1_=*K*_1_/*K*_1_’). In this regard, it should be noted that differences in whole cerebellum CBF (a commonly used reference tissue) have been reported when comparing AD dementia patients and age-matched controls [28]. In contrast, such differences have not been demonstrated for cerebellar cortex CBF by studies using a similar design in terms of technique and participants [24, 29–31]. This suggests that careful interpretation is required when comparing longitudinal *R*_1_ measurement between AD  dementia patients and controls or that alternative reference tissues, unaffected by CBF changes, should be considered. Yet, further research is required to understand whether such changes in cerebellar CBF also occur in early AD stages.

## Conclusion

Relative tracer delivery rate *R*_1_ of [^11^C]PiB showed high global and regional precision in participants covering the AD spectrum. Therefore, [^11^C]PiB *R*_1_ appears to be a stable parameter for measuring cross-sectional differences and longitudinal changes in relative CBF.
